# A Comprehensive Prospective Clinical Study of Hydatid Disease

**DOI:** 10.1155/2014/514757

**Published:** 2014-03-09

**Authors:** Ankit Kayal, Akhlak Hussain

**Affiliations:** ^1^SMS Medical College, Jaipur 302004, India; ^2^Safdarjang Hospital, New Delhi 110029, India

## Abstract

The actual prevalence of hydatid disease in northern part of India is found more than usually interpreted. The present study has been done on 25 patients suffering from hydatid disease of various sites and treated during June 2009 to November 2011 at JLN Medical College and Hospital, Ajmer, with the aim of studying the clinical manifestations of hydatid disease of different sites and/or organ system and of analysing the morbidity and mortality of hydatid disease. The age, sex, h/o dog contact, duration of hospital stay, clinical presentation, treatment advised, findings and difficulties encountered during operation, and postoperative management of patients as well as morbidity and mortality were recorded and analysed. We observed that the mean age was 40 years. The sex incidence revealed female preponderance in the study (M : F: 1 : 2). Duration of illness in the present study varied from 1 month to 6 years in case of liver hydatid disease. Majority of patients were from rural areas (21) and the remaining (4) from urban areas. Swelling was the most common presenting feature. Incidence of hydatid disease at unusual sites in India is higher than in other parts of the world.

## 1. Introduction

Hydatid disease is endemic mainly in the Mediterranean countries (particularly Greece), the Middle East, the Baltic areas, South America, India, northern China, and other sheep-raising areas; however, owing to increased travel and tourism all over the world, it can be found anywhere, even in developed countries [1]. In India, hydatid disease is common in most of the states of which Andhra Pradesh and Tamil Nadu predominate [2]. The liver is the most common site for hydatid disease (75% of cases), followed by lungs (15%), spleen (5%), and other organs (5%) [3]. The diagnosis of hydatid disease is based on epidemiological background of patients, clinical grounds, or noninvasive screening procedures. Chest and abdominal X-ray, ultrasonography, and CT scan should identify cysts characteristics and the diagnosis is confirmed by detecting specific antibodies (immunodiagnostic test). Intradermal Casoni test, the human basophil degradation test, and the complement fixation test have only historical relevance [4]. Only albendazole drug is ovicidal, larvicidal, and vermicidal [5]. Surgery is the gold standard in the management of hydatid cyst liver and other sites as well [6]. With this background, we decided to study the presenting symptomatology and various clinical manifestations of hydatid disease of different sites and/or organ system and to analyse the morbidity and mortality of hydatid disease.

## 2. Material and Methods

The present study has been done on 25 patients suffering from hydatid disease of various sites and treated during June 2009 to November 2011 at JLN Medical College and Hospital, Ajmer. Only those cases were included in whom diagnosis had been confirmed by morphological examination of operative specimen showing either daughter cysts or characteristic white laminated membrane, later confirmed by finding of scolices and laminated membrane under the microscope. When a cyst of peripheral organ/rare site was found on operation, a search for cyst of liver and lung was done by physical examination and/or skiagram of chest to find out any associated cyst in these organs. The age, sex, h/o dog contact, duration of hospital stay, clinical presentation, treatment advised, findings and difficulties encountered during operation, and postoperative management of patients as well as morbidity and mortality were recorded and analysed.

## 3. Results

Majority of the patients belonged to fourth, fifth, and sixth decade of life. The mean age was 40 years. The sex incidence revealed female preponderance in the study (M : F: 1 : 2). Duration of illness in the present study varied from 1 month to 6 years in case of liver hydatid disease. More than 50% of the cases presented within second year of the onset of their illness. Majority of patients were from rural areas (21) and remaining (4) were from urban areas. Among the patients of rural areas, 8 patients were farmers, 7 housewives, 5 labourers, and one student. Out of 4 urban areas patients, 3 were housewives and one was student. History of contact with dogs was available in seven cases.

**Figure 1:**
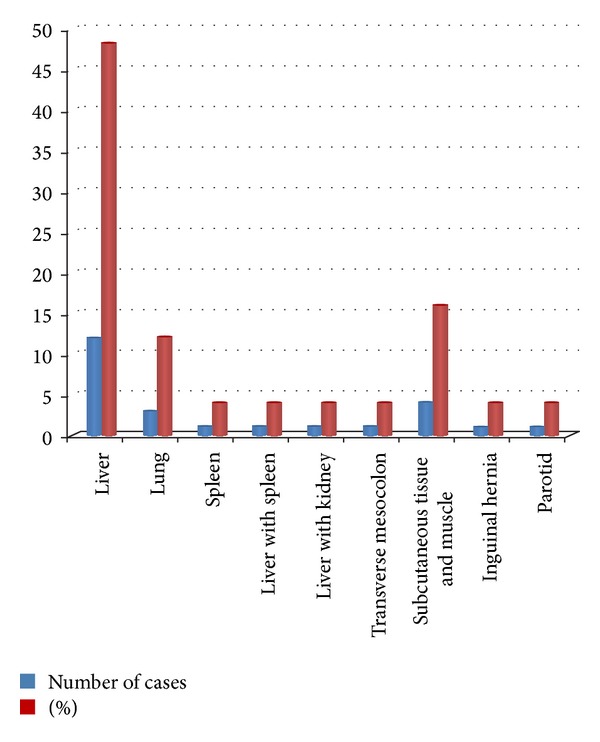
Site distribution of hydatidosis.

**Figure 2:**
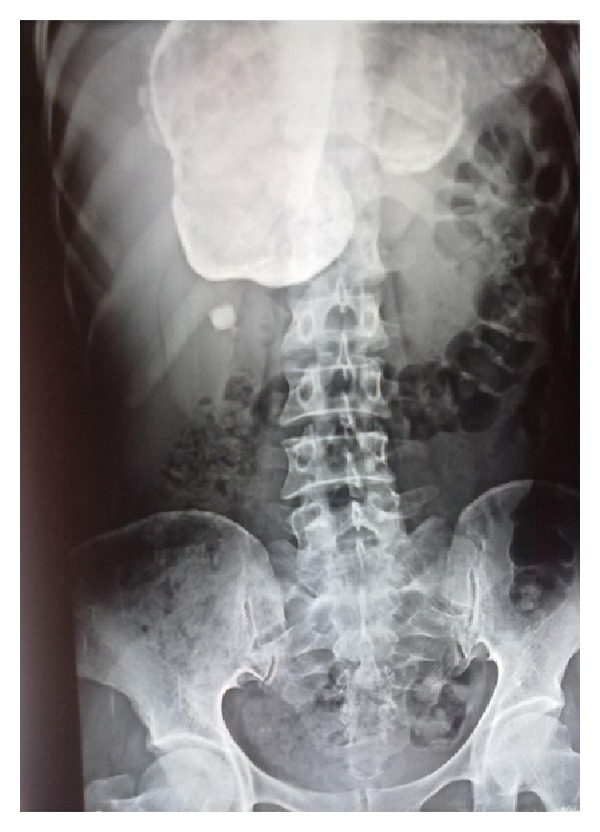
X-ray showing calcified hydatid cyst of liver.

In liver hydatidosis, lump in upper abdomen was the most common presenting feature (Figure 1). One of the patients presented with a tender lump occupying the right upper quadrant with accompanying guarding and rigidity and was diagnosed as amoebic liver abscess; another case was associated with ascites. Cough with expectoration was the usual presenting feature in pulmonary hydatidosis and one of the patients was presented with scanty haemoptysis (Table 1). Differential leucocyte count was performed in all patients. Eosinophilia was present only in 4 patients out of 25 patients. A rim of calcification on plain skiagram of abdomen was present in one case of liver hydatid (Figure 2); in the remaining cases, a soft tissue shadow was evident. In a patient of hydatid cyst in superior surface of right lobe of liver, an elevation of right dome of diaphragm was seen in the chest X-ray film. Intact pulmonary cysts were seen as rounded homogenous and spherical shadows in a chest film. Most of the cysts in liver were present in the right lobe that too on its inferior surface and more than one cyst in liver was present in 3 cases. Maximum number of cysts in liver was 3. In 2 cases, liver was conjointly involved with other viscera. Solitary pulmonary cysts were present in all 3 cases and all were in right side. In cases of subcutaneous tissue and muscle involvement, cysts were distributed as paravertebral region (1), thigh region (2), and anterior abdominal wall (1). Parotid hydatid cyst was present in right parotid region below and anterior to pinna.

14 patients underwent excision or enucleation. Postoperative complications were wound sepsis (5 cases) and biliary fistula (1 case), which prolonged the hospital stay up to 21 days. One patient of pulmonary hydatid disease developed tension pneumothorax due to blockade of tube on the second postoperative period. No death occurred.

## 4. Discussion

As India is not a major sheep-rearing country, it seems that this disease is uncommon here. However, published series mostly from south India and reports of isolated cases from every state of the country indicate that hydatid disease is quite prevalent here. The present study is a clinical study of hydatid disease in central Rajasthan. It is evident from the present study that about 8–10 cases of hydatid disease involving various sites are seen every year in this institution. However reports from south India reveal quite high incidence of this disease. The foreign literature has emphasized strong connection of hydatid disease with sheep-raising industry and the dogs which act as intermediate and definitive hosts, respectively. In the present study, although most of the patients were from rural areas associated with farming and field work and kept domestic animals including goat and sheep as such none was involved in sheep-raising as an occupation. A history of direct contact with dogs was available in six cases; it is thus believed that those patients may have acquired the disease either by transitory handling of dogs or by eating raw vegetables and drinking water contaminated with excreta of an infected dog; a similar view has also been expressed by previous studies [7–10]. Various studies have reported mean age of nearly 40 years which is similar to our study [11–13]. Our study shows sex incidence of about 1 : 2 (M : F) which is close to Ahmet et al. (1999) and Metin et al. (2002) study but differs from that of Palanivelu et al. (about 5 : 1).

In present study, the incidence of liver disease was higher than pulmonary both in adults and children. Similar observations were made by Krishnamurthy and Dalal et al. [14, 15]. The incidence of liver involvement varies from 22.54 to 71.8% and that of lung involvement varies from 4.4 to 48% in these series. Some studies revealed a higher incidence of lung involvement over liver [16, 17], while Trivedi and Navavaty have reported equal distribution between liver and lungs [18, 19]. The low incidence of pulmonary hydatid disease in the initial reports may be due to lack of facilities of thoracic surgery at that time. In the present study, the distribution of liver hydatid was right lobe 11 (78.5%), left lobe 1 (7%), and both lobes 2 cases (14.5%). Maingot in 2004 also reported involvement of right lobe of liver in about 75% of the cases possibly on account of greater blood supply to right lobe than left lobe of liver. Majority of hepatic hydatid cysts are present on the inferior surface and are clinically palpable; the pathognomonic feature of “hydatid thrill” is rarely manifested [20]. Hepatic cysts are slow growing and usually manifest after achieving sufficient size. In this study, hydatid presented as an upper abdominal lump local examination revealed a tense mass in connection with liver. A left sided cyst tends to extend in front of stomach and colon and may be shown by barium examination as distortion or displacement of stomach, duodenum, or colon [20]. Dew (1928) noticed that compensatory hypertrophy occurs in one or other lobes of liver in association with replacing cyst [4]. This was observed in one case of the present study, where left lobe was remarkably hypertrophied and the right lobe was almost completely replaced by cyst. In the present study, 14 cases of liver hydatid disease underwent procedures, namely, excision of endocyst and drainage (10), marsupialization, (3) and omentopexy (1). Drainage procedure and marsupialization were associated with wound sepsis and biliary fistula formations, prolonging the postoperative hospital stay up to more than 21 days. Omentopexy and primary closure gave satisfactory results.

Eosinophilia may be seen in approximately 50% of cases but may be present in other parasitic infestations. In this study, eosinophilia was in 3 patients (12%).

In our study, ultrasonography was proved diagnostic in all cases. In the study conducted by Balik et al. (1999), ultrasonography showed diagnostic accuracy of 97.7%. In our study, CECT scan was done in 12 (48%) cases. It was 100% diagnostic. In the study conducted by Balik et al. (1999), CECT scan showed diagnostic accuracy of 100% [11]. MRI can be helpful in identifying the rim and differentiating this diagnosis from other encapsulated liver lesions. Irregularities of the rim border, which can be accepted as signs of partial detachment, are more reliably demonstrated with MRI than with CT or US [21]. MRI of muscle was done in one of our cases.

In the lungs, about 60% of cysts are said to occur in right lung predominantly in lower lobes which may be due to scolices being inhaled directly [22], while Sudarshan et al. reported that more involvement of left lung occurs predominantly in upper lobes than in right lung [9]. Pulmonary cysts are extraordinarily latent and only about one-quarter is discovered before the onset of some complication which is most commonly respiratory infection so that cough with sputum and pain in chest are the usual presenting symptoms. The detection of recognizable hydatid elements in the sputum clinches the diagnosis; a pleural effusion may contain both hydatid material and marked eosinophilia in the fluid [23]. All 3 patients of pulmonary hydatid disease in the present study presented with cough with expectoration. One patient had minimal haemoptysis; no patient gave history of expectoration of hydatid material. Haemoptysis of small or large quantity may be due to vascular inflammatory reaction and secondary to infection. Roentgen study is of more help in diagnosis of pulmonary hydatids than liver. On chest film, an intact pulmonary hydatid cyst appears as rounded homogenous shadow with well-defined margins.

The incidence of hydatid cyst of spleen varies widely. Four cases of hydatid cyst affecting the spleen were seen in the present study, an incidence of 8%. The abdomen honeycomb appearance due to calcified trabeculae on plain skiagram of abdomen is characteristic of echinococcus alveolaris. In one case, isolated splenic hydatid cyst was present and in another case splenic hydatid cyst was associated with liver cyst also. Single case of hydatid disease affecting the kidney was seen in this study, an incidence of 4%. About 20% of all renal hydatids are single without involvement of any other organs. In present study, renal hydatid was associated with liver hydatid cyst and no hydronephrosis or excision of cyst was done completely. Hydatiduria pathognomonic of renal hydatid disease was not present. Primary hydatid cyst of omentum is very rare. Omentum is usually secondarily involved following rupture of liver hydatid cyst, Maingot 2004 [20]. These omental cysts may present as direct inguinal hernia content later on, as one of cases in the study presented as inguinal region hydatid cyst. The patient was got operated for hepatic hydatid cyst 3 years back than probable due to spillage and developed omental cysts which subsequently present as right inguinal hernial content. It is possible to enucleate these cysts completely. The parotid gland hydatid cysts are always primary (Saxena et al. 1983) [24]. In our study, superficial parotidectomy was done. On follow-up, the patient developed facial palsy which was fully recovered. Sigmoid mesocolonic hydatid cysts are usually secondary to liver cyst. In our study, hydatid cyst of primary transverse mesocolon was found in which enucleation was done.

## 5. Conclusion

1. Incidence of hydatid disease at unusual sites in India (as also reported by other authors from India) is higher than in other parts of the world.
2. As there is no direct evidence of contact with cattle, sheep, and dogs, however, possibility of cases coming in direct contact with these animals during early childhood cannot be ruled out. As most of cases are from rural settings it seems that here hydatid infection takes place by eating raw vegetables and drinking water contaminated by the of infected dogs.
3. A possibility of hydatid disease should be considered in the differential diagnosis of cystic swellings present anywhere in the body especially at rare and unusual sites. In every case of undiagnosed abdominal lump, a possibility of hydatid cyst should be kept in mind.
4. Laboratory methods are not of much help in diagnosis because (i) eosinophilia being nonspecific may be due to other parasitic infestations quite common in this part of the country and (ii) Casoni's intradermal test is usually not done either due to nonavailablity of fresh antigen or due to cases of hydatid cysts of rare sites as this possibility is not suspected.
5. Early treatment is mandatory to avoid local general complications which are directly related to duration of cyst. Aim of treatment is complete removal of parasite without any spillage during operation and unnecessary damage to host tissue.

It is suggested that cooperation of the people, proper checking of the animals, which are slaughtered, and getting rid of stray dogs may help in controlling this disease. Importance of protected water supply, proper cooking of foods, and abstinence from eating raw vegetables which are contaminated obviously prevents the hydatid disease. This message should be carried to the masses especially those living in rural areas through media by lectures, practical demonstration, and radio talks.

## Figures and Tables

**Table 1:** Showing mode of presentation.

Site/organ	Presenting features	Number of cases
Liver	Lump-abdomen	11
	Hepatomegaly	1
	Liver abscess	1
	Splenomegaly	1
	Ascites	1
	Bimanual palpable kidney	1

Lung	Cough with expectoration	2
	Haemoptysis	1

Spleen	Splenomegaly	1

Subcutaneous tissue	Cystic swelling	3

Tissue and muscle	Cold abscess	1

Parotid	Cystic swelling	1

Inguinal canal	Direct hernia	1

Transverse mesocolon	Lump-abdomen	1
